# Zinc Fortification Decreases ZIP1 Gene Expression of Some Adolescent Females with Appropriate Plasma Zinc Levels

**DOI:** 10.3390/nu6062229

**Published:** 2014-06-11

**Authors:** Rosa O. Méndez, Alejandra Santiago, Gloria Yepiz-Plascencia, Alma B. Peregrino-Uriarte, Ana M. Calderón de la Barca, Hugo S. García

**Affiliations:** 1 Research Center for Food and Development (CIAD), Km 0.6 a La Victoria, Hermosillo, Sonora 83304, Mexico; E-Mails: alex_files206@hotmail.com (A.S.); gyepiz@ciad.mx (G.Y.-P.); almabper@ciad.mx (A.B.P.-U.); amc@ciad.mx (A.M.C.B.); 2 Technological Institute of Veracruz, UNIDA, Calzada Miguel Ángel de Quevedo 2779, Veracruz, Veracruz 91897, Mexico; E-Mail: hsgarcia@itver.edu.mx

**Keywords:** adolescent girls, zinc fortified milk, zinc intake, plasma zinc, zinc transporter expression

## Abstract

Zinc homeostasis is achieved after intake variation by changes in the expression levels of zinc transporters. The aim of this study was to evaluate dietary intake (by 24-h recall), absorption, plasma zinc (by absorption spectrophotometry) and the expression levels (by quantitative PCR), of the transporters ZIP1 (zinc importer) and ZnT1 (zinc exporter) in peripheral white blood cells from 24 adolescent girls before and after drinking zinc-fortified milk for 27 day. Zinc intake increased (*p* < 0.001) from 10.5 ± 3.9 mg/day to 17.6 ± 4.4 mg/day, and its estimated absorption from 3.1 ± 1.2 to 5.3 ± 1.3 mg/day. Mean plasma zinc concentration remained unchanged (*p* > 0.05) near 150 µg/dL, but increased by 31 µg/dL (*p* < 0.05) for 6/24 adolescents (group A) and decreased by 25 µg/dL (*p* < 0.05) for other 6/24 adolescents (group B). Expression of ZIP1 in blood leukocytes was reduced 1.4-fold (*p* < 0.006) in group A, while for the expression of ZnT1 there was no difference after intervention (*p* = 0.39). An increase of dietary zinc after 27-days consumption of fortified-milk did not increase (*p* > 0.05) the plasma level of adolescent girls but for 6/24 participants from group A in spite of the formerly appropriation, which cellular zinc uptake decreased as assessed by reduction of the expression of ZIP1.

## 1. Introduction

Zinc is an essential trace element for humans. Its physiological demands are very high in adolescents that are in risk of deficiency, especially if their diets are based on cereals and legumes with high content of zinc absorption inhibitors, as in deprived populations [1]. Zinc deficiency affects growth, sexual maturity, and the immune system [2]. Therefore, it is necessary to reduce risk and one possibility for doing so is to use fortification of highly consumed foodstuffs or food ingredients [3,4].

In Mexico, there is a program for fortification with zinc and other micronutrients in all commercial wheat and corn flours as well as in milk only for targeted groups. We found previously that zinc-fortified milk was effective in increasing both, intake and plasma zinc levels of adolescent girls from the northwest area of the country [5]. Under basal conditions, 35% of the studied adolescents did not meet their zinc requirements and were facing a borderline deficiency.

There are few studies in humans about the control of zinc homeostasis after high or low supplementary levels of zinc in deprived or in borderline deficient populations [6,7]. The objective of the Mexican supplementation programs is to protect individuals with high deficiencies and do not affect those with adequate intake. Therefore, it is important to understand the relationship between zinc dietary intake by fortification and its uptake and excretion, which drives the homeostatic regulation. After intake variations, the control of homeostasis can be affected by changes in the expression of zinc transporters [8]. Some zinc transporters are tissue specific and maintain intracellular zinc concentrations in a narrow physiological range. The ZnT family of transporters decreases cytoplasmic zinc concentrations by secretion, sequestration, or efflux, whereas the ZIP family increases cytoplasmic zinc by influx or release of stored zinc [9]. Therefore, it is likely that the expression of the ZnT genes would be up-regulated, whereas the ZIP genes could be down-regulated in response to the increase of dietary zinc intake. The aim of this study was to evaluate the changes in zinc dietary intake, absorption and plasma concentrations, as well as to investigate the expression levels of ZnT1 and ZIP1 in peripheral white blood cells from 24 adolescent girls after drinking 500 mL/day of milk fortified with zinc and other micronutrients, for 27 days.

## 2. Experimental Section

### 2.1. Subjects

Twenty-four apparently healthy adolescent women (12–16 years) from public high schools in Hermosillo, Sonora, Mexico, participated in this study during the winter of 2011. Data regarding chronological age and age at menarche were collected. Weight and height of the girls were obtained before the first morning of feeding zinc-fortified milk, on admission, and at the end of the study. Body mass index (BMI) was estimated using the Anthroplus software (Version 3.2.2; World Health Organization, Geneva, Switzerland) [10]. Exclusion criteria included pregnancy, use of vitamin and mineral supplements, diarrhea, or other known malabsorption syndromes. At least one of the parents of each participant provided written consent. All procedures were approved by the Ethical Review Committee of the Research Center for Food and Development (CIAD).

### 2.2. Intervention

The intervention consisted of consuming a regular self-selected diet for 27 days, in which the usual milk was replaced by zinc-fortified milk (500 mL/day), which provided 6.6 mg Zn/day. Participant girls received 2 packets of powdered milk weekly and instructions to prepare it with the spoon and cups provided.

### 2.3. Blood Samples

Two blood samples were obtained from fasting participants (5-mL syringe with EDTA). One was used to obtain plasma by centrifugation at 1400× *g* for 10 min at 4 °C. Plasma was separated from the red blood cells and frozen at −70 °C for later total zinc analysis and C-Reactive Protein (CRP). The second blood sample was used to separate leukocytes and measure expression levels of ZnT1 and ZIP1, using real time-RT-qPCR.

#### 2.3.1. Plasma Zinc and CRP Status

The concentrations were quantified by atomic absorption using a Spectr AA-20 (Varian Technotron Pty Ltd., Mulgrave, VIC, Australia) [11]. Zinc standards containing 0.1, 0.5, 1.0 and 1.5 ppm zinc in 1% HCl were used to prepare the calibration curve. The quantitation procedure was validated using non-fat dry milk (NIST, 1549) (NIST, Gaithersburg, MD, USA). CRP was quantified because of the relation between elevated acute phase protein concentrations and low plasma zinc. The amount of CRP was determined by enzyme-linked immunosorbent assays, according to manufacturer instructions (HS-CRP ELISA, DRG, South San Francisco, CA, USA).

#### 2.3.2. Primer Design and Gene Expression Analysis

Expression of zinc transporters (ZIP1 and ZNT1) in the isolated leukocytes was evaluated from two subsamples of six adolescent girls each, the first subsample, group A, was formed by girls whose plasma zinc concentrations increased after the intervention assay, while the second group, group B, was the girls whose plasma zinc decreased after the intervention for 27 days. Measurements were done in blood samples before and after the 27 days trial.

To evaluate gene expression, leukocyte RNA was extracted and purified according to the manufacturer’s instructions (QiaAmp RNA blood mini kit, Qiagen, Hilden, Germany). The amount of RNA was determined by absorbance at 260 nm, and its integrity was assessed by agarose gel-electrophoresis. The primers for ZnT1, ZIP1 and beta-actin (constitutive gene), were designed using the Primer3 software and human sequences from GenBank, and synthesized by Integrated DNA Technologies (San Diego, CA, USA). The primers sequences used are shown in Table 1.

**Table 1:** Primers used for relative gene expression analysis.

Primers		PCR length (bp)	Sequence 5′–3′	Accession No.
ZIP1	Fw	231	CGTGCCTGTGTACTGGTGTT	NM_020342.2
	Rv		ATGACACCTCTAGGCATCGG	
ZnT1	Fw	210	TGGAGGTGGCTAAAACCATT	NM_021194.2
	Rv		TGCTAACTGCTGGGGTCTTT	
Β-actin	Fw	221	GCAAGCAGGAGTATGACG	NM_001101.3
	Rv		GTCACCTTCACCGTTCCAGT	

The specificity of the primers was tested by conventional PCR followed by agarose gel electrophoresis and melting curve analyses after real time PCR. The amplicons were thoroughly sequenced to confirm the identity.

The cDNA was synthesized, in duplicate, from 278 ng of total RNA using the Qiagen QuantiTect^®^ Reverse Transcription (Qiagen). Each reaction for quantitative PCR contained 10 μL 2× SYBR Green Supermix (iQTM SYBR Green Supermix, Bio-Rad, Hercules, CA, USA), 0.7 μM of each primer, 1 μL template DNA (equivalent to 13.93 ng total RNA), and 7.6 μL water. The quantitative PCR reactions were performed on an iQTM5 Real Time PCR Detection System (Bio-Rad). Standard curves of ZIP1, ZnT1 and beta-actin were run to determine the efficiency of amplification. Changes in relative expression were calculated as follows: ΔΔCt = ΔCtq − ΔCtcb, where Ct is the cycle number at which amplification rise above the background threshold, ΔCt is the change in Ct between 2 test samples (initial and final), q is the target gene, and cb is the calibrator gene. Fold change in relative gene expression was then calculated as 2^−ΔΔCt^ [12].

### 2.4. Food Intake

Food intake was assessed on 2 separate days by the method of 24-h recall. Estimation of energy and nutrients intake was done using our laboratory database [13]. Zinc in fortified milk was quantified by atomic absorption. Digestion of food samples was done using a commercial oven (model MDS-2000, CEM Corp, Mathews, NC, USA). The National Institute of Standards and Technology bovine liver (SRM 1577b) and non-fat milk powder (SRM 1549) were used as standards.

The daily molar ratio of phytate:zinc was calculated as follows:

Zn = (mg phytate/660)/(mg Zn/65.4)(1)

### 2.5. Physical Activity Questionnaire

A seven-day physical activity questionnaire was answered by the adolescents that included all their daily physical activities. Values were assigned as multiples of the basal metabolic rate [14] Sedentary < 1.56 mMB, moderate 1.57–1.64 mMB, and heavy 1.65–1.82 mMB.

### 2.6. Socioeconomic Status

It was determined based on personal information such as educational level of the adolescent girls, job status of the household head, and several household belongings [15].

### 2.7. Statistics

The NCSS 2007 software (Number Cruncher Statistical System, Windows version; Kaysville, UT, USA) was used for the statistical analysis. The Kolmogorov-Smirnov test was used to check the normality of the data. Data are presented as means ± standard deviation (SD). Results for the expression of zinc transporters (ZIP1 and ZnT1) in the blood leukocytes isolated were analyzed using a paired Student’s *t*-test (before and after zinc supplementation). Differences were considered significant at *p* < 0.05.

Associations were explored by stepwise multiple regression, where fold change of ZIP1 and ZnT1 were the dependent variables. The independent variables considered in the models were: physical activity level, body mass index, plasma zinc levels, dietary zinc intake, menarche, and days after menstruation when blood was extracted.

## 3. Results

### 3.1. Baseline Characteristics of the Participants

The baseline characteristics of the 24 adolescent girls (Table 2) fell within normal values and were similar (*p* > 0.05) to values measured after the 27-days intervention. However, at baseline, 4 of the girls were overweight, 2 of them were obese and 3 were underweight. After the intervention, 14 girls increased their weight, although their BMI was still in the basal range. The majority of the girls were considered as sedentary, and only 5 of them reported moderate activity.

**Table 2:** Basal characteristics of the 24 adolescent girls participating in the study.

Characteristics	Mean ± SD	Range
Age (year)	14.1 ± 1.1	12–16
Weight (kg)	56.3 ± 11.1	43–77.5
Height (cm)	159.3 ± 4.3	152.5–169.4
BMI/age (kg/m^2^)	22.2 ± 4.3	18.1–32.9
Age at menarche (year)	12.2 ± 1.2	10–14
Physical activity (mMB)	Sedentary	<1.56 mBM

mBM, multiples of basal metabolism. BMI, body mass index.

### 3.2. Intake and Plasma Zinc Changes

Table 3 shows data on dietary intake. There were no significant differences for energy, protein and phytate intake between the basal and final measurements. Mean intake of zinc was higher (*p* < 0.001) after intervention than at the basal conditions, and therefore, similar trends were found for estimated zinc absorption (*p* < 0.05), for the 24 participant girls.

**Table 3:** Mean dietary intake of energy, protein, zinc, phytate and other parameters in study participants.

Variable	Basal	After intervention	*p*
**Energy (kcal/day)**	1930.1 ± 709.5	2193.1 ± 693.8	NS
**Protein (g/day)**	65.15 ± 26.8	73.7 ± 21.0	NS
**Zinc (mg/day)**	10.5 ± 3.9	17.6 ± 4.4	<0.001
**Phytate (mg/day)**	1105.5 ± 1110.5	1384.7 ± 1101.9	NS
**Phytate:zinc molar ratio**	11.6 ± 9.7	6.8 ± 5.4	<0.05
**Estimated Zn absorption**	3.2 ± 1.2	5.3 ± 1.3	<0.001

Mean ± SD. NS: not significant. Statistical significance was *p* < 0.05, *n* = 24.

**Figure 1:**
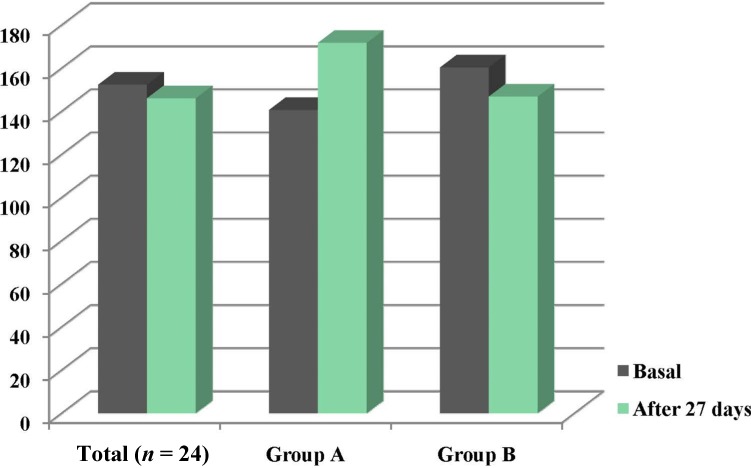
Plasma zinc levels of the total sample, group A and group B at basal condition and after 27 days intervention. * *p* < 0.005, ** *p* < 0.02, NS: non-significant (Student’s *t* test).

In all cases, normal CRP values (<10 mg/L) were observed at baseline and at the end of the study. Figure 1 shows the basal mean values of the plasma zinc concentration which were not changed significantly after 27 days of fortified-milk intake, for the 24 participants (152.4 ± 20.9 *vs.* 146.2 ± 25.7 µg/dL). However, individual plasma concentrations were variable with higher values (*p* = 0.005) after the intervention than baseline in group A (140.7 ± 16 *vs.* 171.8 ± 21 µg/dL), opposed to the other 6/24 adolescents (group B), whose mean concentration was lower (*p* = 0.002) after the 27 days of fortification (170.2 ± 21 *vs.* 145.2 ± 24 µg/dL, respectively).

Plasma zinc concentration was significantly correlated (*p* < 0.05) with the initial and final BMI measurements (*r* = −0.81). According to the Institute of Medicine (U.S.) [16] all of the plasma concentration values were normal (≥70 µg/dL) before the intervention. The increased plasma zinc of adolescents from group A was related to the fortified milk intake. Their zinc intake increased significantly from the baseline (11.53 mg/day) until the end of the intervention (16.02 mg/day). Despite the zinc intake increased from 9.0 ± 3 to 17.1 ± 5 mg/day, their plasma zinc levels were not increased in girls from group B, along the intervention period.

### 3.3. Changes in the ZnT1 and ZIP1 Expression Levels

To understand how the increase of dietary zinc intake was controlled in the body of the group A adolescents who increased the mean plasma zinc concentrations, as well as the ones of group B with decreased plasma zinc, their ZIP1 and ZnT1 gene expression was measured and the results are shown in Table 4.

**Table 4:** Expression of zinc importer (ZIP1) and zinc exporter (ZnT1) in blood leukocytes from adolescent girls from groups A and B, at basal and after 27 day intervention.

Study Group	Basal	After intervention	*p* *
*Fold change of ZIP1*			
**A**	1	−1.40	0.006
**B**	1	−1.44	0.078
*Fold change of ZnT1*			
**A**	1	1.75	0.55
**B**	1	1.09	0.88

Fold change in gene expression of ZIP1 and ZnT1 was normalized with β-actin and calculated as 2^−ΔΔCt^; 1 *=* no change in initial sample; * Student paired *t* test; *p* = statistical significance.

For group A, ZIP1 gene expression in blood leukocytes was reduced 1.40-fold (*p* = 0.006), and this change was related to days after menstruation (*r*^2^ = 0.6631; *p* = 0.04). The gene expression of the exporter ZnT1 did not differ between basal and after zinc intervention (*p* = 0.55). For group B with decreased plasma zinc after the intervention, the ZIP1 expression was reduced by the same value than for group A; however, because of the individual variability, it was not significantly different from the baseline level (*p* = 0.078). For group B, the ZnT1 expression was not affected by the intake of zinc-fortified milk for 27 days (*p* = 0.88).

## 4. Discussion

The characteristics of the 24 adolescent girls in this intervention were almost the same of those reported in our former study [5], except by the mean age (14.1 instead of 15.1 years old). However, there were no differences in the mean plasma zinc levels of adolescent girls in the current study, after the 27 days intervention, while in our former study zinc levels increased after drinking fortified milk in the same period. According to Andree *et al.* [6] and Aydemir *et al.* [17], plasma zinc levels are not the best markers for zinc status because these values would not reflect changes in zinc intakes since they are under tight homeostatic control. However, a transient increase in plasma zinc occurs during supplementation and it may be more evident if the status is initially low [18].

Mocchegiani *et al.* [19] supplemented, using 10 mg/d zinc during 48 days, old European subjects selected on the basis of low plasma zinc levels and IL-6-174 polymorphism (G/C or C+, and G/G or C− genotypes). G/G genotypes are associated with an impaired zinc status. Since zinc parameters as NK cell cytotoxicity, nitric oxide releasing and zinc erythrocyte increased in all the supplemented groups (plasma zinc ≤ 10.5 µM), including C− with unstable plasma zinc. So that the sole assessment of plasma zinc level is not a reliable indicator of zinc status at least for old people. Mariani *et al.* [20] suggest that the potential interaction among circulating zinc increments, changes in immunological parameters and the interactive influence of +647 MT1a and −174 IL-6 polymorphic alleles, could be important determinants for evaluating the efficacy of zinc treatment and for identifying groups of subjects that can take advantage of therapeutic intervention.

To explain our findings in plasma zinc changes between both interventions using the same zinc-fortified milk for 27 days, it is important to consider the basal proportion of adolescents not achieving their requirement (EAR = 7.3 mg/day) for zinc that was 35.2% at the former study and at the present study such proportion was 16.6%. In addition, it can be related to the diet, where phytate presence and protein intake may influence zinc absorption [21], although estimated zinc absorption appear to be similar for both studies (≈3 at baseline and ≈5 at the end of the study, respectively). In the present study done in the winter, participants consumed the same amount of phytate, 14% more energy and 20% more protein than in the former study that was made during the summer and autumn. It has been shown that higher energy and protein intakes in north-western Mexican women are mainly taken between winter and spring compared with summer and autumn [22], and this pattern is related to the extreme desert weather.

It is obvious that the Mexican north-western adolescent population in this study had a wide variability in zinc intake and absorption enhancers. According to a Mexican national survey [23], the highest zinc intake in adolescent girls was in the studied region with respect to the rest of the country.

On the other hand, plasma zinc concentration was negatively related to BMI in group A of the present study. As it is known, zinc metabolism is altered in obesity [24]. In México, obesity and overweight are a public health problem, with 35.8% of the adolescent girls suffering them [25], with similar prevalence for girls in our study area [26]. Overweight people present low serum zinc levels [27], and zinc content of erythrocytes is inversely related to BMI in obese women [28]. Therefore, the high prevalence of overweight and obesity in the studied demographic area, together to insufficient zinc intake and high content of dietary phytates, could be negative factors for the zinc nutritional status of adolescent girls.

Therefore, it could be expected to find a wider variation in zinc status for the general Mexican population, with the same zinc fortification of food ingredients from the national program. Thus, it is very important to understand the relationship between zinc dietary intake by zinc-fortified milk with zinc uptake and excretion, which drive the regulation. The obtained information could be useful to provide feedback for the national fortification program if necessary.

We measured the mRNA expression of two zinc transporters of peripheral blood cells from adolescents of groups A and B, as biomarker of zinc status [6,7]. Plasma zinc in group A increased by 31 µg/dL, while in group B, it decreased by 25 µg/dL. As ZIP1 (importer) and ZnT1 (exporter) are among the most abundantly expressed zinc transporters [24,29] and their expressions were down-regulated and up-regulated, respectively, after short periods of zinc supplementation [6,17], we selected these markers for evaluation.

In the adolescents of group A of our study, whose plasma zinc increased after they drank zinc-fortified milk for 27 days, ZIP1 was down-regulated while ZnT1 mRNA was not significantly affected. The same result of decreased ZIP1 and no change in ZnT1 expression was shown in a study with young women after zinc supplementation of 22 mg/day, for 27 days [6]. However, the ZnT1 expression gave maximum accumulations after 2 and 8 days supplementation of 15 mg zinc/d to young healthy men [17]. Our evaluation was done after 27 days of drinking the zinc-fortified milk, when mRNA expression probably was back to its normal level. Additionally, ZnT1 does not fit well with the *modus operandi* of traditional transporters, but it affects zinc homeostasis through regulating L-type calcium channels [30].

The girls in group B whose mean plasma zinc decreased, showed a wide variability in mRNA expression amounts for ZIP1 and ZnT1. Therefore, mean differences between baseline and after supplementation period with zinc-fortified milk, were not significantly different neither for ZIP1 nor for ZnT1. According to Liuzzi *et al.* [31], the ZIP1 transporter shows a high transcript level when zinc intake is low, but when zinc repletion is reached, it rapidly down-regulates its expression to basal conditions. Zinc intake of participants from group B ranged from 9.04 to 17.07 mg/day and none of the participants had zinc intake under the requirement; their expression of mRNA ZIP1 could be in a wide range of responses. Although we found no relationship between the ZIP gene expression with plasma or zinc intake levels, Noh *et al.* [24] suggest that the expression of zinc transporters may be altered in people with obesity, and then affect zinc homeostasis. So, it is possible that the zinc nutritional status is compromised in a high proportion of the Mexican population, including adolescent girls.

## 5. Conclusions

In conclusion, the adolescents population with none deficiency in zinc, after drinking the zinc-fortified milk for 27 days, did not change their mean plasma zinc levels but in a sub-group A (6/24) ZIP1 expression decreased with no changes in ZnT1 expression levels. Therefore, the transient increase in plasma zinc during supplementation could be an acceptable marker of zinc status as it is the fine ZIP1 expression. Finally, the programs for fortification of foods and food ingredients appear to be effective for increasing zinc consumption and therefore zinc status throughout the year, even for adolescent girls with higher requirements.
